# The CIAMIB: a Large and Metabolically Diverse Collection of Inflammation-Associated Bacteria from the Murine Gut

**DOI:** 10.1128/mbio.02949-21

**Published:** 2022-03-10

**Authors:** Erin Oi-Yan Wong, Emma Joan Elizabeth Brownlie, Katharine Michelle Ng, Sornnujah Kathirgamanathan, Feiqiao Brian Yu, Bryan D. Merrill, Kerwyn Casey Huang, Alberto Martin, Carolina Tropini, William Wiley Navarre

**Affiliations:** a Department of Molecular Genetics, University of Toronto, Toronto, Ontario, Canada; b School of Biomedical Engineering, University of British Columbia, Vancouver, Canada; c Department of Microbiology and Immunology, University of British Columbia, Vancouver, Canada; d Chan Zuckerberg Biohub, San Francisco, California, USA; e Department of Microbiology and Immunology, Stanford University of School of Medicine, Stanford, California, USA; f Department of Bioengineering, Stanford University, Stanford, California, USA; g Department of Immunology, University of Toronto, Toronto, Ontario, Canada; h Humans and the Microbiome Program, Canadian Institute for Advanced Research, Toronto, Canada; The University of Texas Southwestern Medical Center at Dallas; University of Hawaii at Manoa

**Keywords:** *Adlercreutzia*, *Atopobiaceae*, Lachnospiraceae, Muribaculaceae, *Prevotella*, gut inflammation, microbial growth, microbiota

## Abstract

Gut inflammation directly impacts the growth and stability of commensal gut microbes and can lead to long-lasting changes in microbiota composition that can prolong or exacerbate disease states. While mouse models are used extensively to investigate the interplay between microbes and the inflamed state, the paucity of cultured mouse gut microbes has hindered efforts to determine causal relationships. To address this issue, we are assembling the Collection of Inflammation-Associated Mouse Intestinal Bacteria (CIAMIB). The initial release of this collection comprises 41 isolates of 39 unique bacterial species, covering 4 phyla and containing 10 previously uncultivated isolates, including 1 novel family and 7 novel genera. The collection significantly expands the number of available Muribaculaceae, Lachnospiraceae, and Coriobacteriaceae isolates and includes microbes from genera associated with inflammation, such as *Prevotella* and Klebsiella. We characterized the growth of CIAMIB isolates across a diverse range of nutritional conditions and predicted their metabolic potential and anaerobic fermentation capacity based on the genomes of these isolates. We also provide the first metabolic analysis of species within the genus *Adlercreutzia*, revealing these representatives to be nitrate-reducing and severely restricted in their ability to grow on carbohydrates. CIAMIB isolates are fully sequenced and available to the scientific community as a powerful tool to study host-microbiota interactions.

## INTRODUCTION

While the vast majority of studies aiming to experimentally dissect interactions between microbes and their mammalian hosts rely on mouse models, the large majority of cultivated gut microbes are derived from humans (1–3). Large-scale culture collections of human gut microbes have advanced our understanding of microbial growth requirements and facilitated investigations of the ways specific microbes influence host physiology (4, 5). While human and lab mice microbiotas are both dominated by species from the phyla Bacteroidota and Bacillota, they differ considerably at lower taxonomic levels and vary widely in the relative abundance of many species (6–8). Hence, the degree to which the mouse and human microbiota are functionally similar is largely unknown. The few studies that have directly addressed this question found that many isolates of human origin do not stably colonize the mouse gut, are rapidly outcompeted by mouse-derived isolates, and differ in their effects on intestinal immune development (9, 10). These differences confound efforts to align findings from lab mice with conventional (undefined) microbiotas to those from gnotobiotic mice colonized with human fecal samples or synthetic communities. Furthermore, widespread but poorly understood and uncontrolled variability across the microbiotas of lab mice from different facilities has been a major source of irreproducibility in mouse models (11, 12). A comprehensive collection of murine-derived microbial isolates will greatly improve the quality and reproducibility of gut microbiota studies conducted in mice, enable a more complete understanding of how human and mouse microbes are and are not comparable, and also allow the field to make sense of decades of prior research.

16S rRNA gene and shotgun metagenomic sequencing efforts have played a key role in characterizing the gut microbiota of lab mice, but the paucity of cultured species limits our understanding of their functions. A substantial proportion of short 16S rRNA gene sequences and metagenome-assembled genomes (MAGs) cannot be taxonomically assigned at the species level using currently available pipelines and databases, complicating both identification and subsequent targeted infection experiments (13–16). Even within a given species, individual strains may not fill the same ecological niche or behave in a functionally equivalent manner. For example, different strains of Bacteroides ovatus promote the production of various levels of immunoglobulin A (17).

To date, there have been two large-scale cultivations of microbes from laboratory mice: the Mouse Intestinal Bacterial Collection (miBC) and the Mouse Gut Microbial Biobank (mGMB). The miBC comprises 76 species isolated from mice from various genetic backgrounds, providers, and laboratories (18). The mGMB comprises 126 bacterial species obtained from *Lep*^ob/ob^ mice, a model for understanding metabolic diseases (19). Together, the miBC and the mGMB account for only ∼44% of total reads identified in the amplicon data set generated by the miBC study at the species level, indicating that considerable work remains to be done to more completely capture the set of microbes that inhabit the murine gut (19).

A major focus of study relates to the roles various gut microbes play in initiating and prolonging the course of intestinal inflammation (20). Inflammation dramatically changes the intestinal environment via host-produced antimicrobial peptides, infiltration of the intestinal lumen by immune cells, iron limitation, and an increase in oxidative stress through immune-mediated production of peroxides, superoxide, and nitric oxide (21–24). Gut microbiota composition during inflammation often shifts away from strict anaerobes and toward oxygen-tolerant microbes like Pseudomonadota that tolerate or even thrive in the inflamed intestinal milieu (25–28). Some gut microbes can trigger inflammation (pathogens), exacerbate or prolong inflammation (pathobionts), persist and ultimately promote recovery and repair of host tissues, or merely act as resilient but neutral bystanders that presumably evolved to withstand the transient but frequent storms of inflammation occurring during the life of their animal hosts.

Here, we report the results of our efforts to cultivate novel bacterial species and strains that thrive or survive in the inflamed mouse intestine, the Collection of Inflammation-Associated Mouse Intestinal Bacteria (CIAMIB). We exploited a previous observation that different cages in our animal facility of interleukin-10-deficient (*IL10*^−/−^) mice and mice deficient in the adenomatous polyposis coli (*Apc*) and MutS homolog 2 (*Msh2*) genes (*Apc*^Min/+^ *Msh2*^−/−^) displayed different degrees of pathology dependent on the composition of their gut microbiota (29–31). These mouse models are commonly used to investigate the relationship between the gut microbiota and intestinal inflammation, a precursor to diseases such as inflammatory bowel disease (IBD) and colorectal cancer (CRC) (32–34). Cohousing experiments demonstrated that these differences were due almost entirely to gut microbiota composition, as the transfer of microbes from affected mice promoted the development of colitis or polyps in cages of previously healthy isogenic mice (29, 30).

The first release from the CIAMIB reported here comprises 39 species and 41 strains, 10 of which represent novel bacterial species that have never previously been isolated or cultured to our knowledge. We sequenced the genomes of all 41 strains and characterized the growth of each species under a wide range of conditions, leveraging genomic information to examine trends in metabolic profiles across bacterial families and for particular species. The CIAMIB will provide a powerful resource to experimentally dissect the role that specific microbes play in health and disease, particularly inflammation.

## RESULTS

### Establishment of a collection of bacteria from the inflamed mouse gut.

The primary goal of this study was to generate a collection of inflammation-associated bacteria from the mouse gut as a resource for the scientific community. At this stage, we did not seek to isolate specific causal microbes or define whether our cultivated microbes are pathogens, pathobionts, or inflammation-resistant bystanders. To this end, cecal and colonic contents were collected from seven mice that either had developed spontaneous colitis (*IL10*^−/−^ mice; four mice total) or polyps (*Apc*^Min/+^ *Msh2*^−/−^, two mice total) or were *Apc*^Min/+^ *Msh2*^+/−^ mice cohoused with *Apc*^Min/+^ *Msh2*^−/−^ mice with polyps (one mouse total). Colonic and cecal contents were plated on 18 types of agar media and incubated under anaerobic, microaerobic (5% O_2_), and aerobic conditions for 3 to 7 days. Both selective and nonselective media were used to try to recover slow-growing, rare, and fastidious bacteria (Fig. S1). Each isolate was restreaked onto solid media twice, with single colonies picked each time, to increase the likelihood that the isolates were pure and clonal.

10.1128/mBio.02949-21.1 FIG S1 Isolation pipeline used to generate the CIAMIB. Download FIG S1, EPS file, 0.3 MB. Copyright © 2022 Wong et al. 2022 Wong et al. https://creativecommons.org/licenses/by/4.0/ This content is distributed under the terms of the Creative Commons Attribution 4.0 International license.

**FIG 1:**
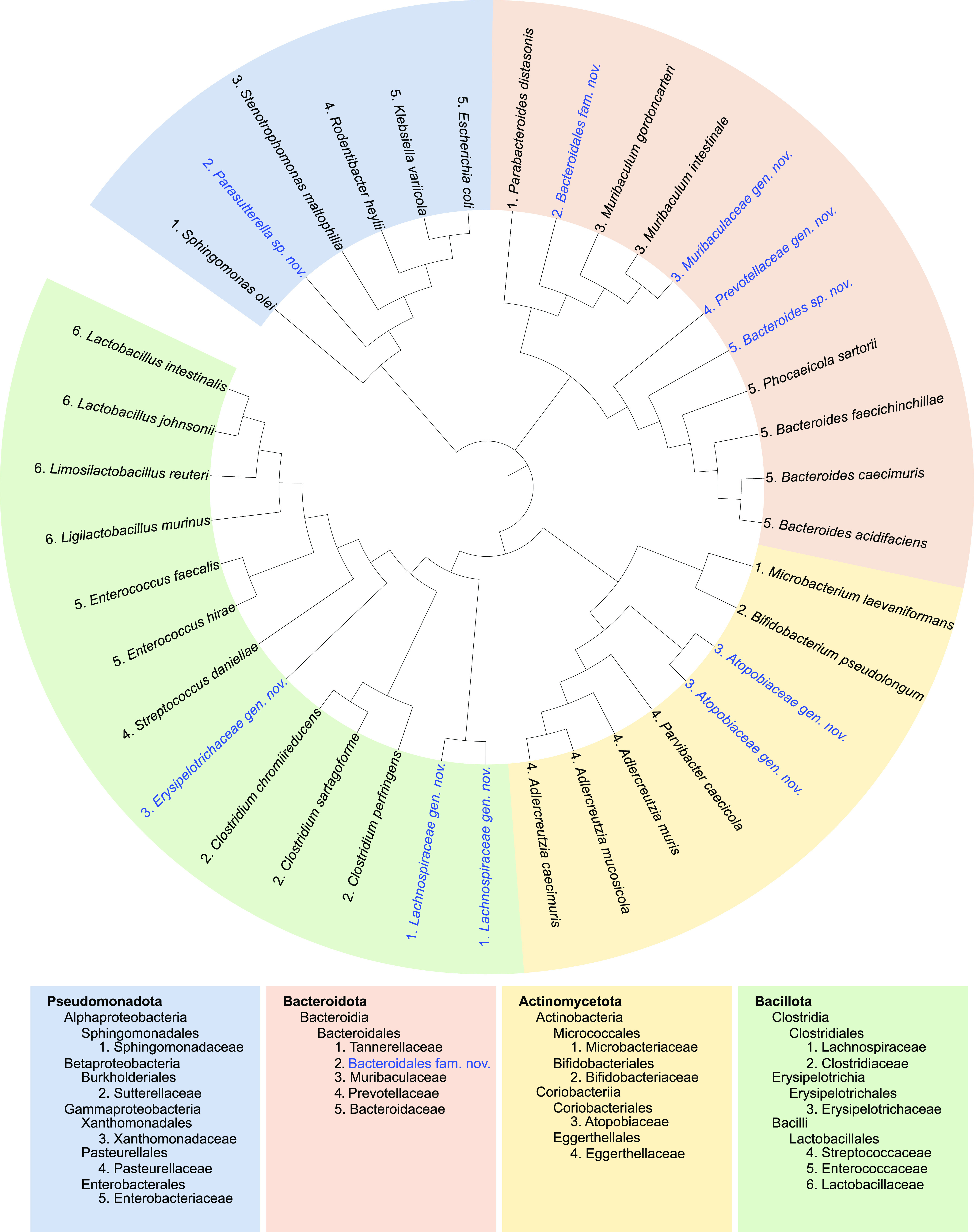
The CIAMIB is phylogenetically diverse. The cladogram illustrates the taxonomic distribution of all 39 species in the CIAMIB based on 16S rRNA gene sequences. Color coding is by phylum, and taxonomy is indicated numerically at the family level. Novel taxa from this study with their candidate names are in blue text.

The identities of 608 isolates were determined by 16S rRNA gene sequencing. Using a sequence identity cutoff of 97% to define species, we identified 39 distinct bacterial species from 18 families, spanning the four major phyla found in human and the mouse gut microbiotas: Bacillota (formerly Firmicutes) (13 species), Bacteroidota (formerly Bacteroidetes) (11 species), Actinomycetota (formerly Actinobacteria) (9 species), and Pseudomonadota (formerly Proteobacteria) (6 species) (Fig. 1). The genomes of the unique isolates were sequenced, and individual draft genomes were generated. Detailed information about each isolate, including isolation conditions, 16S rRNA gene sequences, accession numbers, and assembly statistics, is provided in Table S1.

10.1128/mBio.02949-21.5 TABLE S1 Detailed NCBI and isolation information about CIAMIB isolates. Download Table S1, XLSX file, 0.04 MB. Copyright © 2022 Wong et al. 2022 Wong et al. https://creativecommons.org/licenses/by/4.0/ This content is distributed under the terms of the Creative Commons Attribution 4.0 International license.

The 16S rRNA of 10 species from the CIAMIB had <97% identity to any species cultivated to date. *In silico* DNA-DNA hybridization (DDH) and average nucleotide identity (ANI) comparisons were carried out between the whole-genome sequences of novel taxa and their closest neighboring taxa identified from 16S rRNA gene sequences using the Genome-to-Genome Distance Calculator 2.1 and OrthoANIu calculator (35, 36). The genomes of these 10 isolates were below the species-level DDH threshold of 70% and below the 95% ANI species cutoff, establishing them as novel bacterial species belonging to seven novel genera and one novel family (Table S2) (37). To determine novelty at different taxonomic ranks, we performed a classification analysis using the TypeMat tool available through the Microbial Genomes Atlas (MiGA) web server. This tool estimates the probability of novelty at each rank based on ANI and average amino acid identity (AAI) (Table S2) (38). These novel species have been deposited at the DSMZ to ensure long-term availability (Table S2) and are currently identified by the lowest taxonomic groups to which they can confidently be assigned (e.g., *Bacteroides* [*new species*]).

10.1128/mBio.02949-21.6 TABLE S2 MiGA analysis, *in silico* DNA-DNA hybridization scores and ANI comparisons of novel species against their closest neighboring species identified through 16S rRNA gene identity. Download Table S2, XLSX file, 0.04 MB. Copyright © 2022 Wong et al. 2022 Wong et al. https://creativecommons.org/licenses/by/4.0/ This content is distributed under the terms of the Creative Commons Attribution 4.0 International license.

### Determining novelty of previously uncharacterized taxa in the CIAMIB.

**(i) Novel Bacteroidota.** Microbes within the family Muribaculaceae (previously known as S24-7) have been consistently shown to be highly prevalent in the mouse gut in 16S rRNA gene and metagenomic surveys (39, 40). However, only five isolates from this family have been cultivated to date (10, 18, 40–42). The CIAMIB contains two novel taxa most closely related to Muribaculum intestinale YL27, the first member of the family Muribaculaceae that was cultivated. MiGA classification analysis of isolate NM86_A22 places it in the family Muribaculaceae, potentially within the genus *Muribaculum*. However, the 16S rRNA sequence of NM86_A22 has 90.3% sequence similarity to *M. intestinale* YL27, suggesting that it likely belongs to a novel genus. MiGA classification analysis of the isolates NM04_E33, NM74_B14, and NM76_A32 indicated that these isolates belong to one species in a novel family, corroborated by a 16S rRNA gene sequence similarity of 87.7% to *M. intestinale* YL27.

MiGA classification analysis identified isolate NM73_A23 as a member of the family Prevotellaceae, potentially in a novel genus. By 16S rRNA identity, this isolate is most similar to Prevotella loescheii at 90.2%. Isolate NM69_E16B represents a novel *Bacteroides* species that is most closely related to Bacteroides uniformis (16S rRNA identity of 96.7%).

### (ii) Novel Bacillota.

The CIAMIB contains novel isolates from two families within the phylum Bacillota. Isolates NM01_1-7b and NM72_1-8 were identified by MiGA classification analysis as members of potentially two novel genera within the family Lachnospiraceae, with their 16S rRNA gene sequences most closely matching Ruminococcus gnavus at 91.0% and Murimonas intestini at 92.9% identity, respectively. The Lachnospiraceae are diverse and abundant in the gut but remain poorly represented by available isolates and reference genomes (43, 44). MiGA classification analysis identified isolate NM09_H32 as a member of the family Erysipelotrichaceae, possibly in a novel genus. The 16S rRNA gene sequence of this isolate most closely matched Dubosiella newyorkensis at 90.5% identity (45).

### (iii) Novel Actinomycetota.

Bacteria in the phylum Actinomycetota are ubiquitous in human and mouse gut microbiotas, albeit at a lower abundance than those of the phyla Bacillota and Bacteroidota. Within Actinomycetota, the CIAMIB contains two novel species in the family Atopobiaceae: isolate NM07_P-09 and isolate NM08_P-01. MiGA classification analysis identifies these isolates as likely each belonging to a novel genus. The 16S rRNA gene sequence of NM07_P-09 is most similar to Olsenella umbonata at 92.8% identity, and NM08_P-01 is most similar to Olsenella uli at 94.1%.

### (iv) Novel Pseudomonadota.

Among the Betaproteobacteria class, members of the genus *Parasutterella* are highly prevalent in the human and mouse gut, but only two type strains (Parasutterella excrementihominis and P. secunda) have been cultivated (46, 47). MiGA classification analysis indicates that isolate NM82_D38, whose 16S rRNA gene sequence most closely matches *P. excrementihominis* at 96.0% identity, is a novel species in the genus *Parasutterella*.

### Strain variation within isolates of the same species.

Although 16S rRNA sequencing is frequently used to differentiate species, it is of limited utility in distinguishing between strains within a species. Given that strains can be functionally distinct, we sought to ascertain whether multiple strains coexisted within the same mouse. Of the 608 isolates analyzed, 44% were from either the genus *Bacteroides* or the family Lactobacillaceae. Many isolates were identified as Limosilactobacillus reuteri (formerly Lactobacillus reuteri), Ligilactobacillus murinus (formerly Lactobacillus murinus), or Phocaeicola sartorii.

To determine whether there were substantial genomic differences between these isolates, we employed random amplification of polymorphic DNA (RAPD). RAPD is a molecular technique used to differentiate strains based on PCR amplification of genomic DNA with a random primer, with the resulting amplicons acting as a unique fingerprint for individual strains (48). RAPD was used to examine eight isolates of L. reuteri, nine isolates of *L. murinus*, and five isolates of *P. sartorii* (Fig. S2). Two distinct strains were identified among the eight L. reuteri isolates (CIAMIB strains NM11_1-41 and NM12_1-47) and among the nine *L. murinus* isolates (CIAMIB strains NM26_J9 and NM28_3M-8). The five isolates of *P. sartorii* appeared to be clonal. After verifying the distinct strains by whole-genome sequencing, the CIAMIB was expanded to encompass 41 unique isolates.

10.1128/mBio.02949-21.2 FIG S2 Strain diversity of commonly isolated species. RAPD patterns of *Phocaeicola sartorii*, Limosilactobacillus reuteri, and Ligilactobacillus murinus using 4 sets of primers. Corresponding species and their isolates are listed across the top. Primers for each row are listed on the left. S2188 is a Salmonella enterica serovar Typhimurium strain used as a positive control. Download FIG S2, EPS file, 0.3 MB. Copyright © 2022 Wong et al. 2022 Wong et al. https://creativecommons.org/licenses/by/4.0/ This content is distributed under the terms of the Creative Commons Attribution 4.0 International license.

A comparison of L. reuteri protein-coding genes (as annotated by NCBI) using a 95% amino acid sequence identity threshold revealed that 28.6% of the 2,111 genes in strain NM11_1-41 were not found in NM12_1-47. Conversely, of the 1,924 protein-coding genes in strain NM12_1-47, 21.7% were not found in NM11_1-41. The *L. murinus* strain genomes were similarly distinct, with 24.4% of the 2,305 protein-coding genes in NM26_J9 not found in NM28_3M-8 and 23.6% of the 2,281 genes in NM28_3M-8 not found in NM26_J9 (Table S3). Examples of strain-specific genes include several that encode surface proteins, glycosyltransferases, and flippases, suggesting that the cell surface of each strain is distinct (Table S3).

10.1128/mBio.02949-21.7 TABLE S3 List of Lactobacillus reuteri isolate NM11_1-41 and NM12-1-47 gene functions predicted by KofamScan. Download Table S3, XLSX file, 0.3 MB. Copyright © 2022 Wong et al. 2022 Wong et al. https://creativecommons.org/licenses/by/4.0/ This content is distributed under the terms of the Creative Commons Attribution 4.0 International license.

We also examined the predicted metabolic functions of each strain using the KofamKOALA analysis pipeline provided by the Kyoto Encyclopedia of Genes and Genomes (KEGG) (49). While core metabolisms were largely similar between strains, there were several differences that could be functionally important in the context of the murine gastrointestinal tract. Among the L. reuteri strains, NM11_1-41 uniquely carries genes necessary to synthesize threonine *de novo* from homoserine, while NM12_1-47 uniquely encodes citrate lyase activity (Table S3). NM12_1-47 also encodes acetyl coenzyme A (acetyl-CoA) carboxylase enzymes (*accC* and *accD*) to initiate fatty acid synthesis, a gene cluster that appears to be undergoing loss via pseudogenization and deletion in NM11_1-41. We found that this particular pseudogenization event appears to be common among a large subset of the sequenced L. reuteri isolates available on NCBI, suggesting that subclades of L. reuteri have formed with respect to fatty acid metabolism. Each L. reuteri strain harbors two prophage-like elements that are not found in the other strain, as well as several small genetic islands that are sporadically distributed across the 50 currently sequenced L. reuteri genomes. Differences in core metabolism between the two *L. murinus* strains may be minor and related to the ability to metabolize lactose and rhamnose; many of the genes that differ between NM26_J9 and NM28_3M-8 are currently annotated as hypothetical. NM26_J9 appears to have two prophage-like elements that are absent in NM28_3M-8. Likewise, NM28_3M-8 has a prophage-like genetic element that is absent from NM26_J9.

### Defining the conditions that permit growth of strains in the CIAMIB.

**FIG 2:**
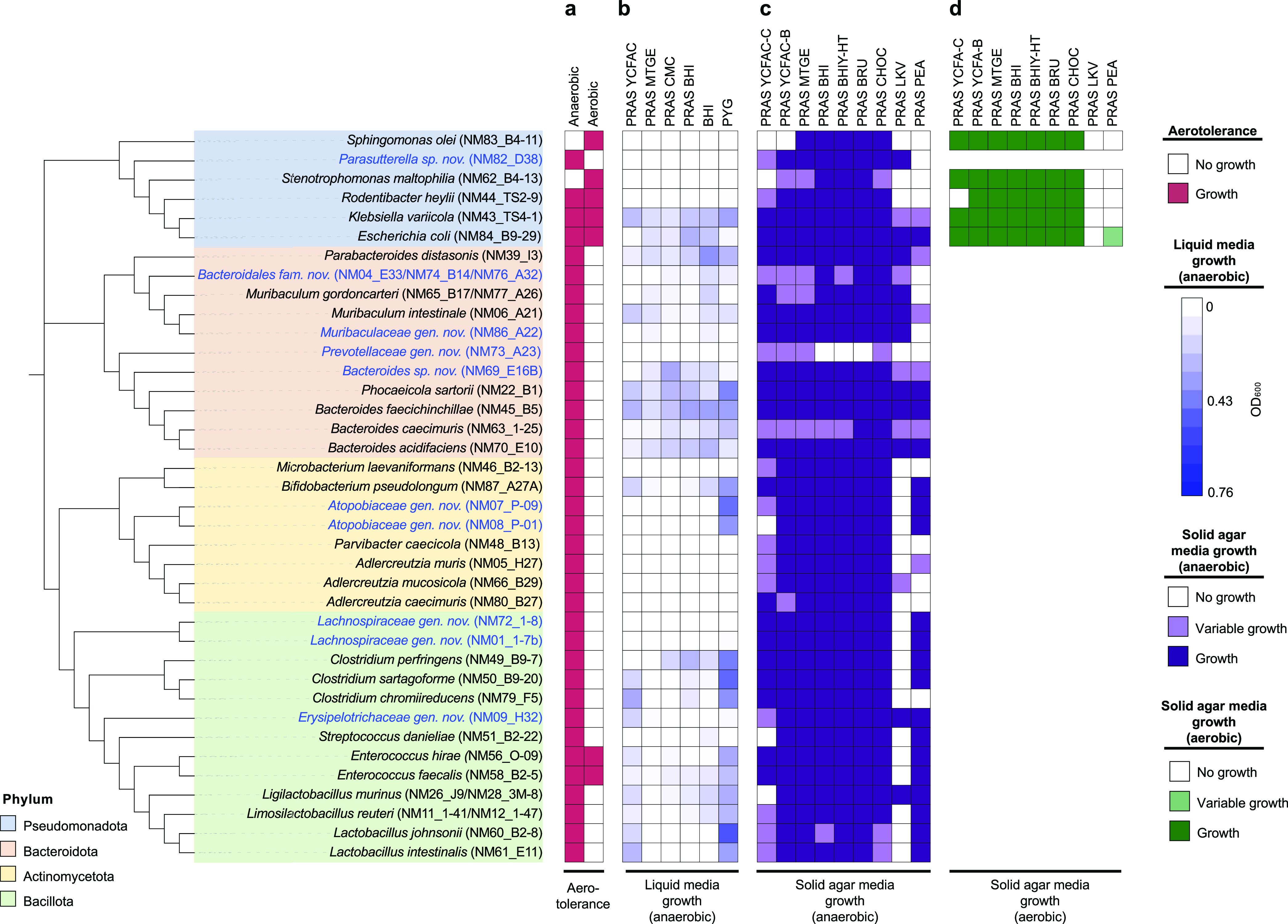
CIAMIB species display diverse growth capacities. The cladogram on the left was generated using 16S rRNA gene sequences and includes all 39 species in the CIAMIB. It is color coded by phylum. Novel taxa with their candidate names are in blue text. From left to right, the growth of each species is shown in different oxygen availabilities (a), liquid media under anaerobic conditions (b), agar media under anaerobic conditions (c), and agar media under aerobic conditions (d). A species was considered to grow in a given agar medium if growth was observed in all three biological replicates. Variable growth was recorded if growth was observed in at least one biological replicate. Liquid media tested include PRAS (prereduced anaerobically sterilized) BHI (brain heart infusion broth), PRAS YCFAC (yeast Casitone fatty acids broth with carbohydrates), PRAS MTGE (anaerobic enrichment broth), PRAS CMC (chopped-meat medium), PYG (peptone yeast glucose broth), and BHI. All solid agar media were PRAS, including LKV (laked Brucella blood agar with kanamycin and vancomycin), PEA (phenylethyl alcohol blood agar), MTGE (anaerobic enrichment agar), BRU (Brucella blood agar), BHIY-HT (BHI agar with horse blood and taurocholate), BHI (BHI agar), YCFAC-B (yeast Casitone fatty acids agar with carbohydrates and sheep blood), YCFAC-C (yeast Casitone fatty acids agar with carbohydrates), and CHOC (chocolate agar).

Gut microbiotas contain many fastidious anaerobic organisms that have complex nutritional and environmental requirements, which makes culturing these species in the laboratory a challenging task. We investigated the ability of each strain from the CIAMIB to grow anaerobically in isolation in five commonly used liquid media and on nine solid-agar media (Fig. 2). Pseudomonadota strains that could be grown aerobically were also assayed for growth under aerobic conditions on the nine solid-agar medium formulations.

Agars that yielded consistent growth in anaerobic or aerobic conditions were Media that Grows Everything - Anaerobic enrichment (MTGE) agar, brain heart infusion (BHI) agar, BHI agar supplemented with horse blood and taurocholate, Brucella agar supplemented with sheep blood, and chocolate agar. Interestingly, many species that could be propagated on a particular solid medium were unable to grow in the same formulation of liquid medium. For example, species belonging to the families Eggerthellaceae and Lachnospiraceae grew poorly in all liquid media tested. This observation may help to explain the poor representation of the family Lachnospiraceae in culture collections, despite its abundance in human and mouse guts (44).

The CIAMIB contains 4 new isolates from the family Muribaculaceae and 3 isolates from a novel, but closely related, family (NM04_E33, NM74_B14, and NM76_A32). Given their prevalence in the guts of many mammals, it is remarkable that so few species in either family have been successfully cultivated. Furthermore, it has been reported that several Muribaculaceae isolates failed to revive after cryogenic storage (40). We hypothesized that these species were more difficult to propagate in liquid media because they require a high inoculum density to resume growth. To evaluate this hypothesis, we tested dilution series for all CIAMIB species within the order Bacteroidales to determine the minimal inoculum optical density necessary for reliable growth initiation. Next, we determined the number of cells required for growth of the two Bacteroidales species that required the highest inoculum (*M. intestinale* isolate NM75_1-48a and isolate NM76_A32) and the lowest inoculum (Bacteroides faecichinchillae isolate NM45_B5 and Parabacteroides distasonis isolate NM39_I3) (Fig. S3A and B). *M. intestinale* and *Bacteroidales* (new family) NM76_A32 required 10-fold and 10,000-fold more cells, respectively, than *B. faecichinchillae* and P. distasonis to reliably resume growth after subculturing. Thus, strategies to cultivate individual bacteria from complex mixtures via limiting dilution may be strongly biased against species that grow poorly in liquid media, or those such as NM76_A32 that grow significantly better only at high cell densities, which may include many Muribaculaceae members.

10.1128/mBio.02949-21.3 FIG S3 Minimum CFUs needed to promote growth of Bacteroidales species in BHI media. (a) Limiting dilution experiment for 4 Bacteroidales species. Cells were resuspended to a standardized OD_600_ of 2 × 10^−2^ and serially diluted 20-fold to determine the lowest inoculation OD that could promote growth in BHI liquid medium. Blue represents growth to saturation in all replicates. Light blue represents growth to saturation in some but not all replicates. Black represents no growth in all replicates. (b) Minimum CFUs required to promote growth in BHI. The number of CFUs inoculated into the last well with growth from panel (a) was determined to ascertain the minimum number of cells required to promote growth. Download FIG S3, EPS file, 0.3 MB. Copyright © 2022 Wong et al. 2022 Wong et al. https://creativecommons.org/licenses/by/4.0/ This content is distributed under the terms of the Creative Commons Attribution 4.0 International license.

### Predicting metabolic profiles of CIAMIB isolates.

We analyzed the draft genome of each CIAMIB isolate to gain insight into its functional metabolic capacity and the considerable differences in growth observed across taxa in the collection. Putative functions were assigned to each predicted protein-coding gene using the KEGG KofamScan pipeline. Strains were clustered on the basis of their functional gene content (Table S4), which revealed that the predicted functional similarity of these isolates approximately, but not perfectly, aligns with their taxonomic relationships. Particularly notable was the clustering of all *Bacteroides* isolates and their separation from Bacillota, Actinomycetota, and Pseudomonadota isolates.

10.1128/mBio.02949-21.8 TABLE S4 CIAMIB isolate gene function predictions by KofamScan. Download Table S4, XLSX file, 0.9 MB. Copyright © 2022 Wong et al. 2022 Wong et al. https://creativecommons.org/licenses/by/4.0/ This content is distributed under the terms of the Creative Commons Attribution 4.0 International license.

The genomes of CIAMIB strains were also bioinformatically assessed using a targeted gut metabolic analysis framework based on a set of manually curated reference modules (50). This analysis searches genomes for the presence/absence of 103 gut metabolic modules (GMMs), each representing a metabolic pathway predefined by a set of orthologous groups involved in the pathway. These GMMs encompass several functions, including catabolism of carbohydrates, amino acids, and lipids; cross-feeding interactions; production of fermentation end products; methanogenesis; and mucus degradation (50).

**FIG 3:**
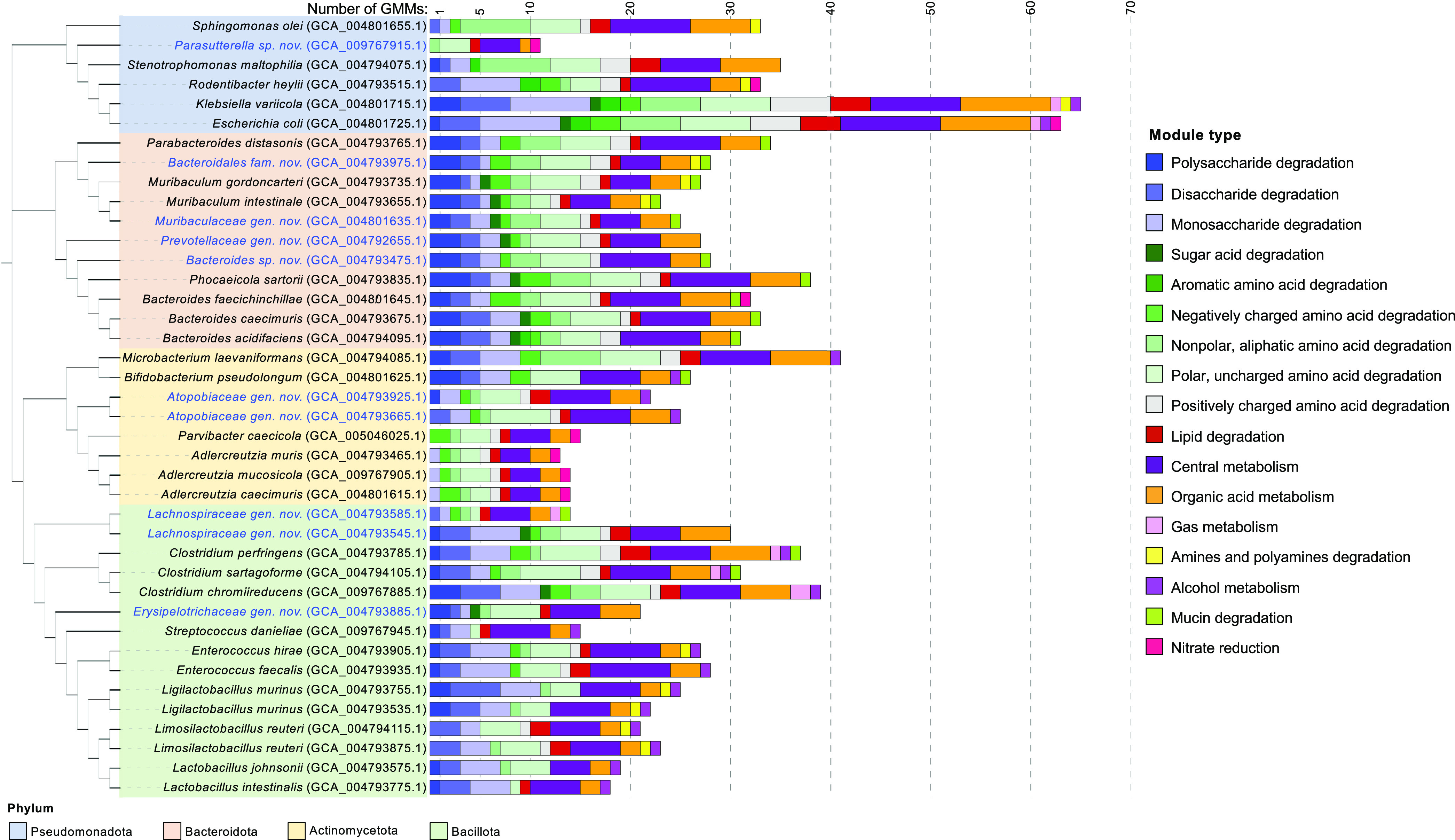
CIAMIB strains display a wide range of metabolic potential. The cladogram on the left shows the taxonomic distribution of CIAMIB strains based on 16S rRNA gene sequences and is color coded by phylum. Gut metabolic modules (GMMs) representing sets of closely related enzymatic functions were identified for all 41 CIAMIB strains.

Bacteroidota species all contained GMMs for polysaccharide degradation, whereas Bacillota were enriched in GMMs for monosaccharide and disaccharide degradation (Fig. 3). These observations align with previous reports on species in the genus *Bacteroides* and the family Lactobacillaceae (51, 52). Many Bacteroidota species contained GMMs for sugar acid degradation, a trait that appeared in only a few species from other phyla, such as the GMM-rich Pseudomonadota members Klebsiella variicola and Escherichia coli. Most species in the CIAMIB contained GMMs to degrade polar and uncharged amino acids, whereas the ability to degrade negatively charged and aromatic amino acids was primarily present in Bacteroidota species, *K. variicola*, and Escherichia fergusonii. Specific GMMs for each strain are detailed in Table S5.

10.1128/mBio.02949-21.9 TABLE S5 Detailed breakdown of GMMs identified in each of the 41 CIAMIB strains. Download Table S5, XLSX file, 0.02 MB. Copyright © 2022 Wong et al. 2022 Wong et al. https://creativecommons.org/licenses/by/4.0/ This content is distributed under the terms of the Creative Commons Attribution 4.0 International license.

Unsurprisingly, isolates differed widely in their predicted metabolic capacities, largely depending on their phylogeny. Isolate NM82_D38 (the novel *Parasutterella* species) had the fewest putative metabolic modules (11 GMMs), while *K. variicola* isolate NM43_TS4-1 contained the most (65 GMMs). The mean across all species was 28 GMMs. However, fastidious obligate anerobic organisms—defined here as obligate anaerobes that failed to reach an optical density at 600 nm (OD_600_) of 0.15 in any liquid medium—had a mean of only 17 GMMs per genome. These findings suggest that fastidious anaerobic organisms fail to grow robustly in liquid culture at least in part due to an inability to synthesize one or more essential metabolites *de novo* or to utilize or access potential sources of essential metabolites in the media.

GMM analysis of species in the family Eggerthellaceae was particularly enlightening, as few complete genomes were previously available and no prior metabolic analysis had been reported. The only carbohydrate degradation module contained in the *Adlercreutzia* genomes (Adlercreutzia muris, A. caecimuris, and A. mucosicola) was for rhamnose. Even more surprising was that Parvibacter caecicola, an Eggerthellaceae species neighboring members of the genus *Adlercreutzia*, contained no carbohydrate degradation GMMs. Analysis of Eggerthellaceae genomes in the CIAMIB for carbohydrate active enzymes (CAZymes) revealed that these isolates contain few glycoside hydrolases, which mediate the hydrolysis of glycosidic bonds, compared to other Actinomycetota from the CIAMIB (Fig. S4) (53, 54). Furthermore, all four Eggerthellaceae species possessed the GMM for nitrate reduction, a feature present in only four other species in the CIAMIB (*B. faecichinchillae* and the Pseudomonadota members E. coli, Rodentibacter heylii, and the novel *Parasutterella* sp.). Our understanding of nitrate-reducing bacteria in the gut microbiota is primarily limited to lactic acid bacteria and enterobacterial pathogens like E. coli and Salmonella (55–57). Nitrate is found at high levels in processed foods and is also a stable end product generated from nitric oxide by activated phagocytes. Phagocyte-derived nitrate can be utilized by gut pathogens and pathobionts as a terminal electron acceptor for anaerobic respiration during inflammation (58). These data suggest that these members of the family Eggerthellaceae represent a novel group of nitrate-reducing members of the gut microbiota that is severely restricted in its ability to grow on carbohydrates.

10.1128/mBio.02949-21.4 FIG S4 Varied prevalence of carbohydrate active enzymes in gut Actinomycetota. Carbohydrate active enzymes (CAZymes) and glycoside hydrolases were identified using the automated CAZyme annotation tool dbCAN2. The total numbers of glycoside hydrolases of each Actinomycetota species in the CIAMIB are shown. Download FIG S4, EPS file, 0.2 MB. Copyright © 2022 Wong et al. 2022 Wong et al. https://creativecommons.org/licenses/by/4.0/ This content is distributed under the terms of the Creative Commons Attribution 4.0 International license.

### Genomes of the CIAMIB strains overlap variably with metagenomic assemblies.

Over the past few years, attempts to assemble complete microbial genomes from shotgun metagenomic sequence data of gut microbiota samples have generated numerous metagenome-assembled genomes (MAGs). A recently published effort, the Integrated Mouse Gut Metagenome Catalog (iMGMC), used data from several independently sequenced and biologically distinct murine gut samples to improve confidence in assigning particular genes to a putative genome (59). The iMGMC effort generated thousands of contigs that were subsequently placed into >1,300 “bins,” where each bin is intended to represent a MAG or the genomic contigs from an individual species or strain. Notably, a significant proportion of the contigs assembled from metagenomic data by the iMGMC were not assignable to a particular MAG and hence were left unbinned.

**FIG 4:**
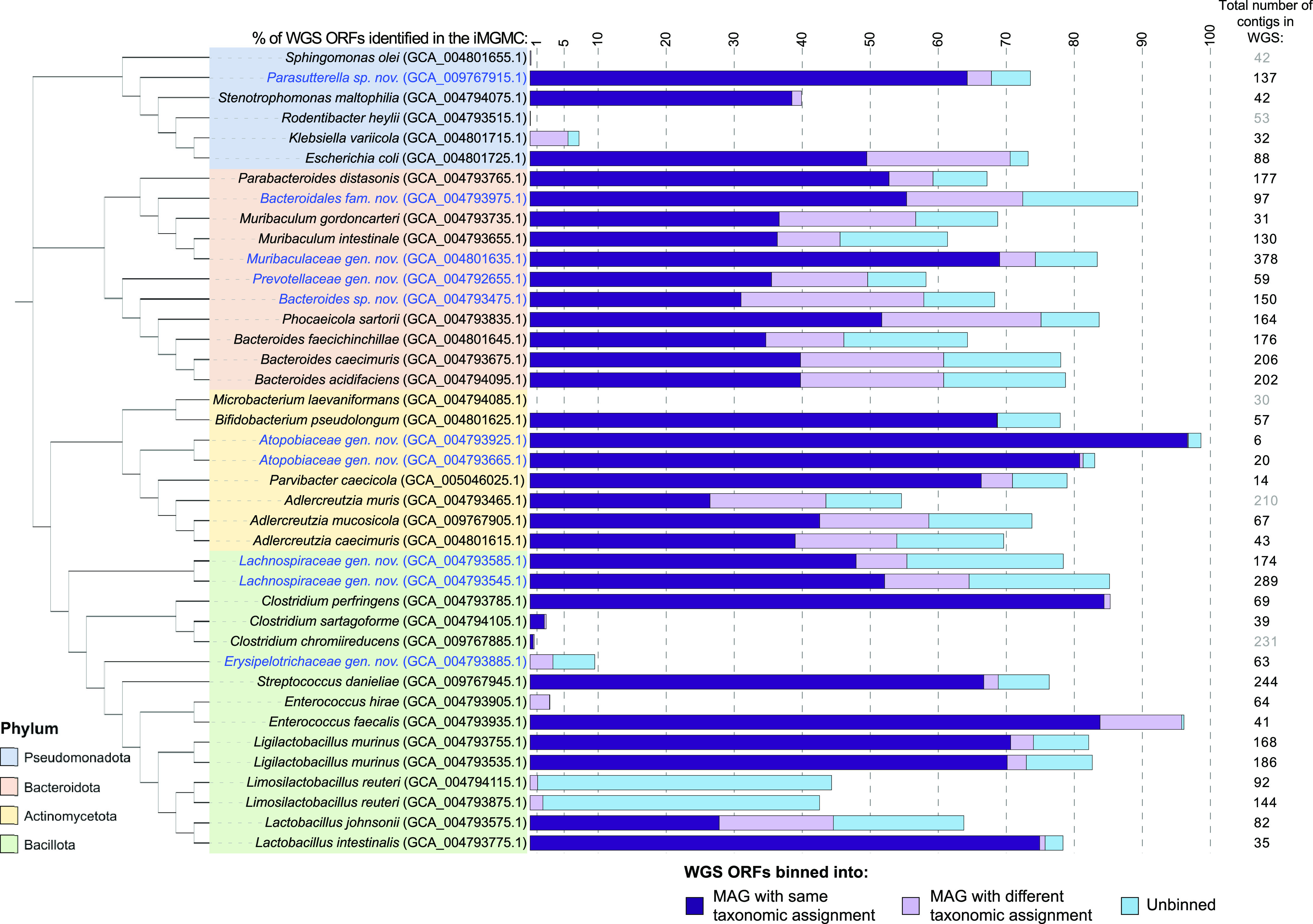
Comparison of CIAMIB ORFs to MAG ORFs. Nucleotide sequences of ORFs identified by the NCBI Prokaryotic Annotation Pipeline from the whole-genome sequences (WGS) of CIAMIB isolates were matched by BLAST (95% sequence identity) to metagenome-derived contigs generated by the Integrated Mouse Gut Metagenome catalog (iMGMC). Shown for each strain are the percentage of total ORFs that were identified in a MAG assigned to the same taxonomic classification as determined by the 16S rRNA gene sequence of CIAMIB isolates (dark purple), a MAG of a different taxonomic assignment (light purple), and to contigs within the iMGMC that were not assigned to a MAG (light blue). The remaining ORFs were not identified among the contigs assembled in the iMGMC. The total number contigs in each assembly is indicated on the far right. Contig numbers in gray represent assemblies in which fewer than 1% of ORFs were identified in the iMGMC.

The genome sequences of our isolates allowed us to retrospectively determine how well MAGs assembled from short-read metagenomic data performed in terms of correct and complete assignments to known strains. Nucleotide sequences of open reading frames (ORFs) identified by the NCBI Prokaryotic Genome Annotation Pipeline from the whole-genome sequences of CIAMIB isolates were matched by BLAST (95% sequence identity) to metagenome-derived contigs generated by the iMGMC. We then determined the percentage of CIAMIB ORFs in the iMGMC that were assigned to a bin with the same taxonomic classification as identified by the 16S rRNA gene of the CIAMIB isolate (Table S6). Among the CIAMIB strains with MAGs represented in the iMGMC data, the proportion of binned sequences with the same taxonomic classification as determined by the 16S rRNA gene varied considerably (Fig. 4). For example, 85% of the ORFs in Clostridium perfringens strain NM49_B9-7 were binned by the iMGMC into a single MAG of same predicted taxonomy. Conversely, the genomic sequences from E. coli and the Bacteroidales species within the CIAMIB were less successfully predicted by the iMGMC, with approximately 50% of the protein-coding genes of most isolates found in contigs not assigned to a MAG by the iMGMC. Many ORFs from CIAMIB isolates were assigned to a bin with a phylogeny inconsistent with the one determined by the 16S rRNA gene, or in some cases were simply not represented in the iMGMC metagenomic assemblies at all. Notably, the genomes of 11 CIAMIB isolates were not represented or were very poorly represented in the iMGMC data, suggesting that species similar to these particular isolates were not present in the microbiota of the mice used to generate the data for the iMGMC. These findings confirm the idea that, while useful, most MAGs represent only incomplete and partly accurate genomes of individual strains and that some taxa, like Bacteroidales, have genomes that present a greater challenge to assemble into MAGs than other taxa, like C. perfringens.

10.1128/mBio.02949-21.10 TABLE S6 CIAMIB ORFs compared to iMGMC ORFs. Download Table S6, XLSX file, 0.02 MB. Copyright © 2022 Wong et al. 2022 Wong et al. https://creativecommons.org/licenses/by/4.0/ This content is distributed under the terms of the Creative Commons Attribution 4.0 International license.

## DISCUSSION

Here, we report the first iteration of the CIAMIB, a collection of bacteria from the colons and ceca of mice with colonic inflammation. The CIAMIB contributes to the growing but still limited number of mouse gut isolates available to the research community (18, 19, 60). Future microbiota research will be greatly accelerated by expanding the number of available isolates associated with a variety of diseases, including obesity, diabetes, nonalcoholic fatty liver disease, and neurologic and psychiatric disorders. Our functional characterizations of the CIAMIB isolates, including optimal growth conditions, will enable the construction of defined microbial communities that can be tailored to recapitulate the microbiota during various physiological states. Gnotobiotic mice colonized with a defined microbiota constructed entirely from well-characterized isolates will be an important tool to experimentally define the role that any individual species or strain plays during the host inflammatory response.

Several isolates within the CIAMIB are particularly notable with respect to the role they may play during inflammation. Enterococcus faecalis, Pasteurellaceae species (*R. heylii*), and *K. variicola* have been found at a higher abundance in IBD and CRC patients than healthy cohorts (61–63). We isolated novel species from the genus *Parasutterella* and the family Prevotellaceae, both of whose increased abundance has been associated with several diseases, including IBD (64–66). Our isolate from the family Erysipelotrichaceae is of interest, as there have been numerous reports of the potential of this family to modulate host physiology and/or disease (67–71), including high immunogenicity, increased abundance after treatment with broad-spectrum antibiotics, and enrichment in CRC (45, 67, 68, 72, 73).

Other taxa of note in the CIAMIB include novel Lachnospiraceae and Muribaculaceae species. Both of these families are deficient in taxonomic resolution and cultured isolates despite being diverse and abundant members of the mouse gut microbiota (10, 18, 39, 41). While higher Lachnospiraceae abundance has primarily been associated with a healthy intestinal state, 16S rRNA metagenomic sequencing of our *IL10*^−/−^ mice showed that Lachnospiraceae remained abundant even under inflammation conditions, an observation supported by other studies (30, 62, 74, 75). The limited literature discussing the family Muribaculaceae has demonstrated both increases and decreases during intestinal inflammation and both blooms and depletions following treatment with broad-spectrum antibiotics (28, 76, 77). These conflicting associations remain common in gut microbiota research and are not surprising given the bacterial diversity encompassed at high taxonomic levels.

Metagenomic studies have become increasingly important for understanding the functional potential of a given microbiota and for *in silico* reconstruction of uncultivated bacterial genomes. Our results suggest caution regarding the increasing reliance on correlating specific functions to particular microbiota members based on short-read 16S sequencing surveys, as two species with nearly identical 16S sequences can harbor very different metabolic or pathogenic potential. We also observed that a large fraction of the putative genomes computationally assembled from very large and diverse short-read shotgun metagenomic experiments were neither accurate nor complete. We therefore urge caution against assuming that MAGs represent accurate genome reconstructions of individual strains or species.

Future work will aim to identify and cultivate species that are underrepresented or not represented in current culture collections. One potential bottleneck we identified in isolating individual microbes from a complex sample are cases, such as Muribaculaceae species, in which the microbe will not reliably resume growth when diluted to a single cell. Additionally, in trying to elucidate growth conditions for CIAMIB species, we found that several that could be propagated on a particular agar medium could not be cultured in the liquid medium of the same formulation. It is possible that certain species will be refractory to isolation using dilution-based protocols and might grow only in the presence of other microbes or with the addition of spent culture media (18, 19, 78). Despite the caveats mentioned above, MAGs can be a powerful tool for identifying the metabolic bottlenecks of uncultivated species and can aid in the design of targeted strategies and growth media for their cultivation.

In conclusion, our work provides a foundational collection of inflammation-associated microbes from the mouse gut. The CIAMIB can be used independently or in combination with other collections to define the roles that each microbe plays in the gut ecosystem, in the absence and presence of inflammation. We provide useful information on culturing each species in the collection and report on the growth patterns within related taxa. The collection will facilitate future functional studies and help to refine strategies for targeted isolation as the field works to expand the availability of mouse gut bacterial isolates.

## MATERIALS AND METHODS

### Mouse background and colon sample collection.

All mice used were of the C57BL/6J background. *Apc*^Min/+^ mice were purchased from Jackson Laboratories and carry a nonsense mutation in exon 15 of the *Apc* gene. *IL10*^−/−^ mice and *Msh2*^+/−^ mice were provided by Ken Croitoru and Tak Wah Mak, respectively, of the University of Toronto. *IL10*^−/−^ and *Apc*^Min/+^ *Msh2*^−/−^ mice developed colitis spontaneously without any treatment. *Apc*^Min/+^ *Msh2*^+/−^ mice did not spontaneously develop colitis but harbored a microbiota similar to that of *Apc*^Min/+^ *Msh2*^−/−^ mice based on 16S rRNA gene sequencing. *IL10*^−/−^ mice were raised under conventional conditions and fed a Teklad Global 18% protein rodent chow (Harlan, WI, USA).

Seven- to 9-week-old mice were sacrificed by cervical dislocation and immediately transferred to an anaerobic chamber (Anaerobe Systems AS-580) containing 10% CO_2_, 10% H_2_, and 80% N_2_ (Praxair NI CD10H1C-K). The working area, dissection tools, and abdomen of each mouse were soaked with 70% (vol/vol) ethanol before dissection to avoid contamination. Cecal, mucosal, and colonic contents were collected by scraping the intestinal wall. For each mouse, these contents were weighed and resuspended in prereduced phosphate-buffered saline (PBS) with 0.1% cysteine to a final concentration of 15 mL of PBS per gram of contents. Suspensions were vortexed for 5 min and allowed to settle for 5 min, after which the supernatant was transferred to a new tube and used as starting material. Aliquots of supernatant and supernatant with equal volumes of prereduced 50% glycerol were frozen for subsequent genomic extractions and isolations, respectively. The remaining supernatant was used in isolations.

### Culture media.

All quantities are per 1 L of medium unless otherwise indicated. Postautoclaving reagents were added after the medium cooled to 50°C. Media containing sheep blood were poured immediately, as sheep blood addition cools down agar substantially.

### (i) Stock solutions.

*(a) Vitamin K (1 mg/mL)*. One hundred microliters of vitamin K1 (Sigma-Aldrich V3501) were diluted into 10 mL of 95% ethanol.

*(b) Hemin (5 mg/mL).* Fifty milligrams of hemin (Sigma-Aldrich H9039) were dissolved in 1 mL of 1 M NaOH. The volume was adjusted to 10 mL using distilled water, then filter-sterilized.

*(c) l-Cysteine (500 mg/mL).* Five grams of l-cysteine hydrochloride monohydrate (Sigma-Aldrich C6852) were dissolved in 10 mL distilled water and then filter-sterilized.

### (ii) Types of media used for isolations.

Descriptions of medium are adapted from the work of Lau et al. [44].

*(a) AIA.* We followed the manufacturer’s instructions for Actinomycete isolation agar (AIA; Himedia M490).

*(b) Beef agar.* Beef agar with l-cysteine (0.5 g/L), hemin (10 mg/L), and vitamin K (1 mg/L) was prepared as follows. We followed the manufacturer’s instructions for cooked meat medium (Himedia M149S) with several modifications. Before autoclaving, we added 15 g agar (BD 214010). After autoclaving, we added 1 mL l-cysteine stock solution, 2 mL hemin stock solution, and 1 mL vitamin K stock solution.

*(c) BHI.* For brain heart infusion (BHI) agar with l-cysteine (0.5 g/L), hemin (10 mg/L), and vitamin K (1 mg/L), we followed the manufacturer’s instructions for BHI broth (Oxoid CM1135) with 15 g agar added and several modifications. After autoclaving, we added 1 mL of l-cysteine stock solution, 2 mL of hemin stock solution, and 1 mL vitamin K stock solution.

*(d)* Brucella *agars.* For Brucella agar with sheep blood (5%), hemin (5 mg/L), and vitamin K (1 mg/L), we followed the manufacturer’s instructions for Brucella broth (BD 211088) with several modifications. Before autoclaving, we added 15 g agar (BD 214010). After autoclaving, we added 1 mL hemin stock solution, 1 mL vitamin K stock solution, and 50 mL defibrinated sheep blood (Cedarlane CL2581-100D). For Brucella agar with sheep blood (5%), trimethoprim (5 mg/L), vancomycin (10 mg/L), and polymyxin B (2500 IU/L), we followed the manufacturer’s instructions for Brucella broth (BD 211088) with several modifications. Before autoclaving, we added 15 g agar (BD 214010). After autoclaving, we added 50 mL defibrinated sheep blood (Cedarlane CL2581), 1 mL trimethoprim (Sigma-Aldrich 92131) dissolved to 5 mg/mL in dimethyl sulfoxide (DMSO), 2,500 IU polymyxin B sulfate (Sigma-Aldrich P0972) dissolved in distilled water and filter-sterilized, and 1 mL vancomycin hydrochloride (Sigma-Aldrich V2002) dissolved to 10 mg/mL in distilled water and filter-sterilized.

*(e) Columbia agars.* For Columbia agar with sheep blood (5%), we followed the manufacturer’s instructions for Columbia agar (BD 211124) with one modification. After autoclaving, we added 50 mL defibrinated sheep blood (Cedarlane CL2581). For Columbia agar with colistin and nalidixic acid (CNA), we followed the manufacturer’s instructions for CNA (BD 212104) with one modification. After autoclaving, we added 50 mL defibrinated sheep blood (Cedarlane CL2581).

*(f) Laked blood agar.* For laked blood agar with kanamycin (100 mg/L) and vancomycin (7.5 mg/L), we followed the manufacturer’s instructions for tryptic soy broth (BD 211825) with several modifications. Before autoclaving, we added 5 g yeast extract (BD 212750) and 15 g agar (BD 214010). After autoclaving, we added 10 mL vitamin K stock solution, 1 mL hemin stock solution, 0.8 mL l-cysteine stock solution, 2 mL kanamycin monosulfate (BioShop KAN201) dissolved to 50 mg/mL in distilled water and filter-sterilized, 75 μL vancomycin hydrochloride (Sigma-Aldrich V2002) dissolved to 100 mg/mL in distilled water, and 50 mL laked sheep blood (Cedarlane CL2581-100L).

*(g) LB agar.* We followed the manufacturer’s instructions for Luria-Bertani (LB) agar (BD 244520).

*(h) MacConkey agar.* We followed the manufacturer’s instructions for MacConkey agar (BD 281810).

*(i) Mannitol salt agar.* We followed the manufacturer’s instructions for mannitol salt agar (BD 211407).

*(j) McKay agar.* McKay agar was 13.3 g nutrient broth (BD 234000), 5 g dextrose (BioShop GLU501), 15 g agar (BD 214010), 10 g yeast extract (BD 212750), 5 g tryptone (BD 211699), 2 g K_2_HPO_4_, 40 mL salt solution (10 g/L NaHCO_3_, 2 g/L NaCl, 1 g/L K_2_HPO_4_, 1 g/L KH_2_PO_4_, 0.5 g/L MgSO_4_·7H_2_O, 0.25 g/L CaCl_2_·2 H_2_O), 1 mL Tween 80, 1 mg crystal violet, 60 mg bromocresol purple, 10 μg vitamin K, 0.05 g hemin, and 15 g Bacto agar in 1 L of distilled water. After autoclaving, we added 20 mL l-arginine (2.5% [wt/vol]), sulfadiazine (Sigma-Aldrich S8626), colistin sulfate, and oxolinic acid to final concentrations of 500 mg/L, 10 mg/L, and 5 mg/L, respectively (79).

*(k) Phenylethyl alcohol agar.* For phenylethyl alcohol agar, we followed the manufacturer’s instructions (BD 211539) with one modification. After autoclaving medium, we added 50 mL defibrinated sheep blood (Cedarlane CL2581).

*(l) Reinforced clostridial agar.* We followed the manufacturer’s instructions for reinforced clostridial agar (Oxoid CM0151).

*(m) Rifampin blood agar.* For rifampin blood agar, we followed the manufacturer’s instructions for Brucella broth (BD 211088) with several modifications. Before autoclaving, we added 15 g agar (BD 214010). After autoclaving, we added 50 μL of 1,000 μg/mL rifampin (Sigma R3501) (10 mg dissolved in 2 mL absolute ethyl alcohol, with 8 mL distilled water added), and 50 mL defibrinated sheep blood (80).

*(n) Sulfite polymyxin sulfadiazine agar.* Sulfite polymyxin sulfadiazine agar was 15 g pancreatic digest of casein, 15 g agar (BD 214010), 10 g yeast extract (BD 212750), 0.5 g ferric citrate, 0.5 g Na_2_SO_3_, 0.12 g sulfadiazine (Sigma-Aldrich S8626), and 0.01 g polymyxin sulfate in 1 L of distilled water.

*(o) THB agar.* For Todd-Hewitt broth (THB) agar with yeast extract, we followed the manufacturer’s instructions for THB (BD 249240) with two modifications. Before autoclaving, we added 10 g yeast extract (BD 212750) and 15 g agar (BD 214010).

*(p) Tryptic soy agars.* For tryptic soy agar with sheep blood (5%), we followed the manufacturer’s instructions for tryptic soy broth (BD 211825) with two modifications. Before autoclaving, we added 15 g agar (BD 214010). After autoclaving, we added 50 mL defibrinated sheep blood (Cedarlane CL2581). For tryptic soy yeast agar with sheep blood (5%), l-cysteine (0.5 g/L), hemin (10 mg/L), and vitamin K (1 mg/L), we followed the manufacturer’s instructions for tryptic soy broth (BD 211825) with several modifications. Before autoclaving, we added 3 g yeast extract (BD 212750) and 15 g agar (BD 214010). After autoclaving, we added 50 mL defibrinated sheep blood (Cedarlane CL2581), 1 mL l-cysteine stock solution, 2 mL hemin stock solution, and 1 mL vitamin K stock solution.

### (iii) Media used to propagate CIAMIB isolates after isolation.

Once isolated, most strains were found to grow on Brucella agar supplemented with sheep blood, hemin, and vitamin K (Hardy Diagnostics A30) and were therefore routinely propagated on this medium. The two exceptions were Streptococcus danieliae and Prevotellaceae (new genus) isolate NM73_A23, which were propagated on THB agar with yeast extract and in BHI broth, respectively.

### Isolation and identification of bacterial strains.

Gut sample resuspensions were diluted 10-fold, and 100 μL were plated on 17 types of agar medium (see compositions in “Culture media”). All media were placed in an anaerobic chamber at least 48 h prior to use to remove oxygen. Plates were then incubated at 37°C for 2 to 14 days under the following conditions: aerobic; anaerobic with a mixture of 10% hydrogen, 10% carbon dioxide, and 80% nitrogen; or hypoxic with a mixture of 5% oxygen, 10% carbon dioxide, and 85% nitrogen. Each day, plates were checked for growth, and when single colonies appeared, they were restreaked onto fresh plates. This procedure was always performed at least twice to obtain a single-colony morphology.

Once colonies appeared morphologically pure, several were picked and resuspended in 50 μL sterile distilled water. Samples were boiled for 15 min and then centrifuged for 5 min at 16,000 × *g.* The supernatant was transferred to a new tube, and 5 μL were used as the template DNA for PCR-based amplification of the 16S rRNA gene. PCRs were carried out using the 16S rRNA gene universal primers 8f (5′-AGAGTTTGATCCTGGCTCAG-3′) and 1492r (5′-CGGTTACCTTGTTACGACTT-3′) (81). Amplification was performed using the ProFlex 3 by 32-well PCR system, in 50-μL reaction mixtures with 5 μL of 10× buffer, 1.5 μL of 50 mM MgCl_2_, 1 μL of 10 mM deoxynucleoside triphosphates (dNTPs), 5 μL of 10 mM forward primer, 5 μL of 10 mM reverse primer, 5 μL of DNA template, 27.75 μL of nuclease-free water and 0.25 μL of *Taq* DNA polymerase. The cycler was programmed for 2 min at 94°C for initial denaturation, followed by 32 cycles of 1 min at 94°C for denaturation, 1 min at 56°C for annealing, 2.5 min at 72°C for extension, and finally 10 min at 72°C for the final extension. To check for amplification, PCR products were run at 100 V for 30 min on a 1% (wt/vol) agarose gel containing SYBR Safe DNA gel stain (Invitrogen S33102) diluted to 1/20,000 in 1× Tris-acetate-EDTA (TAE) buffer. Successfully amplified samples were purified using the GeneJET PCR purification kit (Thermo Scientific K0701). Next, Sanger sequencing of the 16S rRNA gene was carried out at The Centre for Applied Genomics (Toronto, Canada) using the 8f primer. Sequences were identified by performing a BLAST search of the 16S rRNA gene sequences against the NCBI reference RNA sequences (refseq_rna) database. A cutoff of 97% 16S rRNA gene identity was used to delineate novel taxa (82).

Cryo-stocks were prepared by resuspending cells grown for 3 to 5 days (depending on colony morphology) from plates in Brucella broth with glycerol (25% vol/vol), and stored at −80°C.

### Bacterial growth assays.

Strains were initially grown anaerobically from frozen stocks on media as described above for propagation at 37°C for 72 h. Strains were then expanded twice in order to obtain enough bacterial cells for assay. For the assay, bacterial cells were resuspended in peptone yeast glucose (PYG) medium (following the instructions for DSMZ medium 104). To test for growth on solid medium, resuspensions were plated separately on various agar media divided into 6 sections. Two microliters of the suspension were deposited into each sector and spread using 1-μL inoculation loops. Afterward, plates under anaerobic conditions were incubated for 72 h at 37°C and plates under aerobic conditions were incubated for 48 h at 37°C. After incubation, plates were observed visually, photographed, and scored as growth (presence of colonies) or no growth (absence of colonies). Assays were performed in biological triplicate. A species was considered to grow in a given medium if growth was observed in all three replicates. Variable growth was recorded if growth was observed in at least one biological replicate. Growth under the various conditions was plotted using iTOL (83).

Solid growth was evaluated on nine types of prereduced, anaerobically sterilized (PRAS) media provided by Anaerobe Systems: laked Brucella blood agar with kanamycin and vancomycin (LKV), phenethyl alcohol blood agar (PEA), MTGE anaerobic enrichment agar (MTGE), Brucella blood agar (BRU), brain heart infusion agar with horse blood and taurocholate (BHIY-HT), brain heart infusion agar (BHI), yeast casitone fatty acids agar with carbohydrates and sheep blood (YCFAC-B), yeast Casitone fatty acids agar with carbohydrates (YCFAC), and chocolate agar (CHOC).

To test for growth in liquid media, resuspensions were diluted to an OD_600_ of 1 and inoculated into 200 μL of each of six types of liquid medium to a starting OD_600_ of 0.1 in a 96-well plate. Liquid cultures were incubated anaerobically for 72 h at 37°C. Wells were mixed every 24 h by pipetting. To measure the final OD_600_, 100 μL of culture from each well were transferred to a new 96-well plate, and the OD was recorded using a Tecan 96-well plate reader (Infinite 200 Pro). Assays were performed in technical duplicate and biological triplicate. Raw OD_600_ values were first averaged between technical replicates, normalized by subtracting the medium blank to correct for medium turbidity, and averaged between three biological replicates. Growth under various conditions was plotted using iTOL (83).

Liquid growth was evaluated in six types of broth (four PRAS media provided by Anaerobes Systems and two media made in house and reduced before use): BHI, YCFA, MTGE, chopped-meat medium with carbohydrates, BHI with 10 mg/L hemin and 1 mg/L vitamin K (Oxoid), and PYG.

To determine the minimum number of cells needed to promote growth in BHI for four of the *Bacteroidales* species, strains were initially grown anaerobically from frozen stocks on Brucella blood agar with hemin and vitamin K (Hardy Diagnostics A30) at 37°C for 48 h. Strains were then expanded by streaking twice more on Brucella blood agar to obtain enough bacterial cells for assay. For the assay, cells were resuspended to a standardized OD_600_ of 0.02 and serially diluted 20-fold in a 96-well plate. Five microliters from each dilution were also spotted onto Brucella agar supplemented with sheep blood, hemin, and vitamin K and incubated anaerobically at 37°C for 72 h to determine the concentration of the initial resuspension in CFU per milliliter. This concentration was used to calculate the starting CFU in each well. Ninety-six-well plates were incubated anaerobically at 37°C for 48 h and mixed by pipetting at 24 h. To measure the final OD_600_, 100 μL of each dilution were transferred to a new 96-well plate, and the OD was recorded using a Tecan 96-well plate reader (Infinite 200 Pro). The assay was performed in technical duplicate and biological triplicate. Raw OD_600_ values were first averaged between technical replicates and then normalized by subtracting the medium blank to correct for medium turbidity. A minimum OD_600_ of 0.1 was used to determine successful growth. The number of CFU inoculated into the last well with successful growth in BHI was determined to be the minimum number of cells needed to promote growth. The minimum number of cells needed to promote growth was averaged across three biological replicates.

### Whole-genome sequencing.

In this study, 46 draft genomes were constructed, representing each strain in the CIAMIB and all Muribaculaceae isolates. DNA sequencing was carried out by the Genomics Platform of the Chan Zuckerberg Biohub or performed in house (detailed in NCBI BioSample information).

Genomic DNA for most isolates was extracted using the DNeasy PowerSoil kit (Qiagen 12888) following manufacturer’s instructions. *S. danieliae* genomic DNA was extracted using the PureLink genomic DNA minikit (Invitrogen K182001) with several modifications. Briefly, *S. danieliae* was streaked onto two full THB agar plates and incubated anaerobically at 37°C for 72 h. Cells were collected and resuspended in tubes containing 200 μL TE (10 mM Tris, 1 mM EDTA; pH 8), 500 U/mL mutanolysin, and 15 mg/mL lysozyme and then incubated with gentle shaking for 4 h at 37°C. Next, 25 μL of 28.8 mg/mL proteinase K were added, and cells were incubated for 1 h at 56°C. Finally, 265 μL of PureLink genomic lysis/binding buffer were added, and tubes were briefly vortexed before incubation at 55°C for 30 min. The remaining steps followed the manufacturer’s instructions for binding, washing, and eluting DNA. Genomic DNA was eluted with 50 μL UltraPure DNase/RNase-free distilled water.

All DNA libraries were prepared for sequencing using the Nextera XT DNA library preparation kit (Illumina FC-131) following the manufacturer’s instructions. In-house sequencing was carried out using an Illumina MiniSeq according to manufacturer’s instructions. At the Chan Zuckerberg Biohub, Nextera XT libraries with final volumes of 4 μL were constructed using a miniaturized and high-throughput process leveraging low-volume liquid handlers, including a Mantis (Formulatrix), a Mosquito X1, and a Mosquito HTS (SPT Labtech). Libraries were pooled and sequenced on an Illumina NovaSeq S4 flow cell.

Reads were assembled using the comprehensive genome analysis service provided by the Pathosystems Resource Integration Center (PATRIC) through their Bacterial Bioinformatics Resource Centre (84). This service assembled reads using SPAdes v. 3.10.0 with the activated BayesHammer tool and MismatchCorrector (85, 86). Assemblies were evaluated using QUAST v. 2.3 (87). Predictions and annotations were performed with the NCBI Prokaryotic Genome Annotation Pipeline (88). Genome sequences were submitted to NCBI under the accession numbers provided in Table S1. Assemblies were also evaluated using CheckM (v1.0.18) through the KBase UI (version 2.5.2) platform with the default parameters to determine genome completion and contamination (89, 90).

### Genome-based analyses.

Multiple alignment of the full-length (>1,400-nucleotide [nt]) 16S rRNA gene sequence of each species (using a 97% cutoff value) was performed using Muscle in MEGA7 (91). This alignment was used to construct a phylogenetic tree based on the maximum-likelihood algorithm, using the Tamura-Nei model in MEGA7 (91). The tree was then annotated using iTOL (83).

Taxonomic classification based on average nucleotide identity (ANI) and average amino acid identity (AAI) was performed using the TypeMat tool from the Microbial Genomes Atlas (MiGA) (r2021-04 update). MiGA is a web server that allows the classification of genomes against all taxonomically assigned taxa with available genome sequences in the NCBI RefSeq and NCBI Genome Database, available at http://microbial-genomes.org (38).

Digital DNA-DNA hybridization (dDDH) to ascertain novel taxa was performed through the Genome-to-Genome Distance Calculator (GGDC) 2.1 using formula 2, a web service provided by the DSMZ (http://ggdc.dsmz.de) (35, 36). The genome of each species belonging to a novel taxon was compared to the reference genomes of the three phylogenetically closest species based on 16S rRNA sequence identity (37). The ANI between novel taxa and their closest neighboring species based on 16S rRNA sequence identity was calculated using the OrthoANI algorithm, available at https://www.ezbiocloud.net/tools/ani (92).

To obtain gut metabolic modules (GMMs), the set of functional genes from each strain was first defined using BLAST, filtering out non-coding sequence regions. DNA sequences were then converted into amino acid sequences and annotated using the Kyoto Encyclopedia of Genes and Genomes (KEGG) KofamScan pipeline using their database of prokaryotic hidden Markov models (HMMs) (93). Through this pipeline, KEGG Orthology (KO) numbers were assigned to the predicted coding genes of each bacterial strain on the basis of their similarity to the HMMs. To assess functional relatedness, CIAMIB strains were clustered by the presence/absence of the various KO functions identified using the clustermap function (method = “ward”) of the Seaborn data visualization library based on the Python matplotlib library (94, 95) in a custom Python script.

Separately, the KO lists generated for each strain were used as input into Omixer-RPM using the human GMM database to map KO-annotated genes to predicted metabolic pathways (50, 96), using a module coverage cutoff 66% as indicated in the original publication (50). Total numbers of GMMs identified in each functional group were plotted using iTOL (83).

Carbohydrate-active enzymes were identified using the dbCAN2 meta server, a webserver for automated carbohydrate-active enzyme annotation, using their HMMER (hits: E value < 1e−15, coverage > 0.35), DIAMOND (hits: E value < 1e−102, hits per query [-k] = 1), and Hotpep (hits: frequency > 2.6, hits > 6) tools. Carbohydrate hydrolases identified by two or more tools were quantified and plotted.

For each strain in the CIAMIB, the protein-coding sequences were used to search assembled iMGMC contigs using standalone BLAST (BLASTn) from NCBI with a sequence identity threshold of 90% identity over 90% of the gene sequence. The protein-coding genes in each strain were assigned to specific contigs and, subsequently, MAGs and predicted taxonomic groups based on tables provided by the iMGMC (97). Taxonomic classifications assigned to MAGs by the iMGMC were manually compared to classifications of the 16S rRNA gene sequence of CIAMIB isolates assigned by BLAST (refseq_rna) to determine percentage of ORFs with the same classification. For novel taxa, the comparison was made to the highest taxonomic level of confidence (>94.5% for genus level, >86.5% for family level).

### RAPD.

Six sets of primers were used in RAPD (random amplification of polymorphic DNA) analysis of L. reuteri, *L. murinus*, and *P. sartorii*: GS10 (5′-TGCACGGACG-3′), GS15 (5′-CCCACATCCC-3′), GS16 (5′-CCCAACCACC-3′), GS17 (5′-GGGAAGGAGG-3′), GS19 (5′-GGCACTGACG-3′), and GS20 (5′-TCCACCGACC-3′). Genomic DNA was extracted using the GenElute bacterial genomic DNA kit (Sigma-Aldrich) following the manufacturer’s instructions and diluted to a final concentration of 10 ng/μL. PCR amplification was carried out using a Bio-Rad PCR machine, in a 50-μL reaction mixture with 5 μL of 10× buffer, 1.5 μL of 50 mM MgCl_2_, 1 μL of 10 mM dNTPs, 10 μL of 10 mM primer, 5 μL of DNA template, 27.25 μL of nuclease-free water, and 0.25 μL of *Taq* DNA polymerase (Thermo Fisher). The cycler was programmed for 20 s at 94°C for initial denaturation, followed by 35 cycles of 30 s at 95°C for denaturation, 30 s at 35°C for annealing, 1.5 min at 72°C for extension, and 10 min at 72°C for the final extension. To visualize, PCR products were run at 100 V for 30 min in a 1% (wt/vol) agarose gel containing SYBR Safe DNA gel stain (Invitrogen S33102) diluted 1/20,000 in 1× TAE buffer.

### Data availability.

All genome assemblies are publicly available in NCBI under BioProject accession number PRJNA474907. Genome sequences were submitted to NCBI under the accession numbers provided in Table S1. All novel taxa are available as individual cultures from the German Collection of Microorganisms and Cell Cultures (DSMZ) or upon request.
